# Post-translational modification and conformational state of Heat Shock Protein 90 differentially affect binding of chemically diverse small molecule inhibitors

**DOI:** 10.18632/oncotarget.1099

**Published:** 2013-07-07

**Authors:** Kristin Beebe, Mehdi Mollapour, Bradley Scroggins, Chrisostomos Prodromou, Wanping Xu, Mari Tokita, Tony Taldone, Lester Pullen, Bettina K. Zierer, Min-Jung Lee, Jane Trepel, Johannes Buchner, Daniel Bolon, Gabriela Chiosis, Leonard Neckers

**Affiliations:** ^1^ Urologic Oncology Branch, Center for Cancer Research, National Cancer Institute, Bethesda, MD, USA; ^2^ University of Sussex, John Maynard Smith Building, Falmer, Brighton, UK; ^3^ Molecular Pharmacology and Chemistry Program, Memorial Sloan-Kettering Cancer Center, New York, NY, USA; ^4^ University of Massachusetts Medical School, Department of Biochemistry and Molecular Pharmacology, Worcester, MA, USA; ^5^ Technische Universität München, Department of Chemistry, Garching, Munich, Germany; ^6^ Medical Oncology Branch, Center for Cancer Research, National Cancer Institute, Bethesda, MD, USA; ^7^ Department of Biochemistry and Molecular Biology, SUNY Upstate Medical University, Syracuse, NY, USA; ^8^ Department of Urology, SUNY Upstate Medical University, Syracuse, NY, USA

**Keywords:** Hsp90, posttranslational modification, phosphorylation, drug binding, Hsp90 inhibitor

## Abstract

Heat shock protein 90 (Hsp90) is an essential molecular chaperone in eukaryotes that facilitates the conformational maturation and function of a diverse protein clientele, including aberrant and/or over-expressed proteins that are involved in cancer growth and survival. A role for Hsp90 in supporting the protein homeostasis of cancer cells has buoyed interest in the utility of Hsp90 inhibitors as anti-cancer drugs. Despite the fact that all clinically evaluated Hsp90 inhibitors target an identical nucleotide-binding pocket in the N domain of the chaperone, the precise determinants that affect drug binding in the cellular environment remain unclear, and it is possible that chemically distinct inhibitors may not share similar binding preferences. Here we demonstrate that two chemically unrelated Hsp90 inhibitors, the benzoquinone ansamycin geldanamycin and the purine analog PU-H71, select for overlapping but not identical subpopulations of total cellular Hsp90, even though both inhibitors bind to an amino terminal nucleotide pocket and prevent N domain dimerization. Our data also suggest that PU-H71 is able to access a broader range of N domain undimerized Hsp90 conformations than is geldanamycin and is less affected by Hsp90 phosphorylation, consistent with its broader and more potent anti-tumor activity. A more complete understanding of the impact of the cellular milieu on small molecule inhibitor binding to Hsp90 should facilitate their more effective use in the clinic.

## INTRODUCTION

Normal eukaryotic cells depend on the molecular chaperone heat shock protein 90 (Hsp90) to maintain protein homeostasis [1-2]. Because of their genetic instability and stressful environment, cancer cells display a greater dependence on Hsp90 and other components of the cellular homeostatic machinery. As a result, Hsp90 has become an attractive target for cancer therapy and there are currently 17 chemically distinct Hsp90 inhibitors being evaluated in cancer clinical trials [3]. Promising data from trials evaluating Hsp90 inhibitors have been reported for several cancers, including non-small cell lung cancer and HER2+ breast cancer [4-7].

Hsp90 is a dimer formed from two identical protomers. Each protomer contains three domains: an amino-terminal (N) domain that contains a nucleotide binding pocket shared by ATP and inhibitory drugs, and several co-chaperone (proteins regulating Hsp90 function)-interacting motifs; a middle (M) domain that harbors sites for client interaction and additional co-chaperone binding motifs, and a carboxy-terminal (C) domain that contains a dimerization motif and interaction sites for additional co-chaperones. Upon ATP binding to the N domain, Hsp90 undergoes an ordered series of conformational changes, termed the chaperone cycle, that begin with transient dimerization of the N domains to confer ATPase competence. Coupled with ATP hydrolysis and with the assistance of defined co-chaperones, this chaperone cycle allows Hsp90 to modify client protein conformation/stability [8-10]. Hsp90 inhibitors interfere with this cycle at its early stages by replacing ATP, leading to the regulated ubiquitination and proteasome-mediated degradation of most client proteins [11].

Accumulating evidence suggests that, despite targeting an identical binding site, chemically distinct Hsp90 inhibitors may preferentially recognize and trap various conformational states of the chaperone [12]. While recent analyses utilizing *in vitro* reconstituted systems have shed light on several aspects of Hsp90-inhibitor interaction, such studies do not recapitulate the presentation of Hsp90 in the human cancer cell, including the potentially complex impact of the chaperone's numerous and dynamic posttranslational modifications on drug binding[13-20].

To begin to better understand these parameters, we have investigated the impact of the cellular milieu on the binding preferences and consequences of two chemically unrelated Hsp90 inhibitors, the benzoquinone ansamycin geldanamycin (GA) and the purine analog PU-H71 (PU). Further, we queried the ability of these inhibitors to access similar Hsp90 conformational states. Our data suggest that PU samples a more diverse repertoire of Hsp90 conformations compared to GA, and these differences are amplified in a cellular context. However, both inhibitors prevent the N domain dimerization that is necessary for a productive chaperone cycle. We also find that inhibitor binding is not uniformly affected by Hsp90 phosphorylation. These data suggest that additional posttranslational modifications may differentially affect drug binding and influence their cellular Hsp90 inhibitory activity in ways not predicted by *in vitro* analysis.

## RESULTS AND DISCUSSION

### GA and PU recognize overlapping but not identical cellular Hsp90 populations

**Figure 1:**
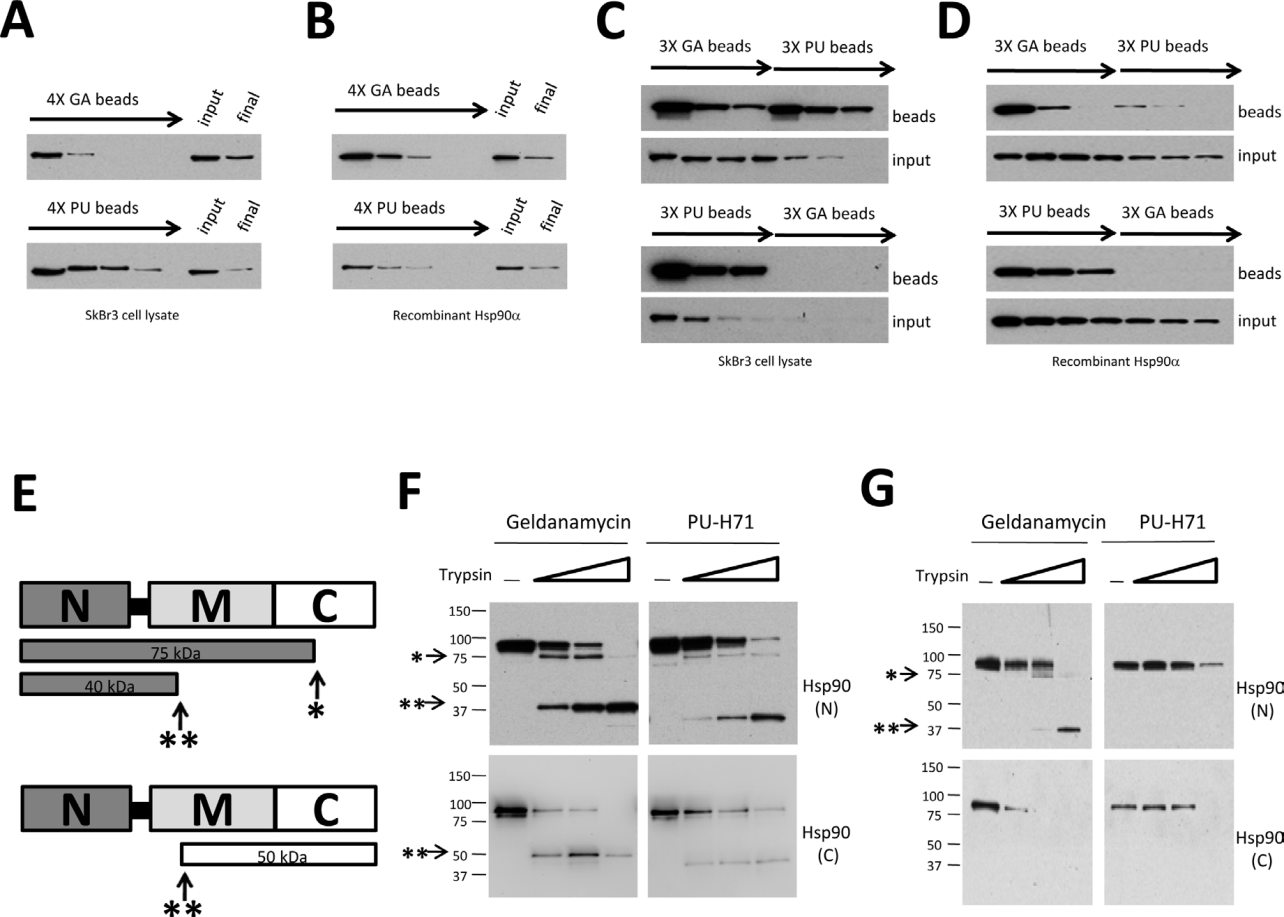
GA and PU recognize overlapping but distinct Hsp90 populations which are not equally sensitive to protease cleavage (A and B) Serial challenge of SkBr3 cell lysate (A) or recombinant Hsp90 protein (B) with GA and PU beads. (C and D) Serial challenge of SkBr3 cell lysate (C) or recombinant Hsp90 (D) with GA-beads followed by PU-beads (top) and vice versa (bottom). The GA-bindable Hsp90 population is contained within a larger PU-bindable population. (E) Cartoon of major tryptic cleavage sites in Hsp90, including a site 75 kD from the N-terminus (*) and another that is 40 kD from the N-terminus (**). (F, G) Purified recombinant Hsp90 (F) or Hsp90 from SkBr3 cell lysate (G) were captured by GA- or PU-beads. Samples were digested with increasing concentrations of trypsin, separated by SDS-PAGE, and subjected to Western blotting with Hsp90 antibodies whose epitopes are either in the N-terminal (N) or C-terminal (C) domains

In order to investigate the binding preferences of GA and PU in cancer cells, we used drug-conjugated agarose as an investigative tool. Consistent with a recent study [12], we found that repeated challenge of a tumor cell protein lysate with GA- or PU-conjugated agarose beads could not capture the entire Hsp90 population, although PU-beads were able to capture a larger fraction of Hsp90 compared to GA-beads (Figure 1A). The unbound Hsp90 fraction retained affinity for Hsp90-specific antibody and ATP-conjugated agarose ([12], and data not shown), suggesting that although it was not accessible to drugs, this population of Hsp90 maintained a native conformation. Repeated challenge of recombinant Hsp90 protein with drug-beads yielded a qualitatively similar result, although the discrepancy between GA- and PU-beads was less apparent (Fig. 1B). To discern whether the Hsp90 populations isolated by each inhibitor were mutually exclusive, we subjected a tumor cell lysate to several rounds of GA-agarose followed by several rounds of PU-agarose, and vice versa. We found that, after depletion of the GA-bindable population, there remained a significant fraction of Hsp90 with affinity for PU (Fig. 1C, top panel). However, the reverse was not true, suggesting that the GA-bindable cellular Hsp90 pool is contained within a more abundant PU-bindable fraction (Fig. 1C, bottom panel). When we performed a similar analysis using recombinant Hsp90 protein, we observed less divergence between GA-bindable and PU-bindable populations (Fig. 1D), suggesting that cell-dependent modifications of Hsp90 contribute to this discrepancy. Treatment of whole cells with excess soluble drug support this interpretation, as PU-agarose was able to capture Hsp90 not quenched by soluble GA, but soluble PU completely inhibited Hsp90 interaction with immobilized GA (data not shown).

Different Hsp90 conformations display characteristic protease sensitivity profiles [21-22]. Therefore, we used trypsin proteolysis to further investigate the state of GA- and PU-bound Hsp90. Previously described major tryptic cleavage sites in the middle (**) and C-terminal (*) domains of Hsp90 [21-22] are shown in Figure 1E. After initial affinity pulldown of purified recombinant Hsp90 protein with drug-bound agarose, we incubated samples with increasing concentrations of trypsin and we visualized the resultant protein fragments by Western blotting with Hsp90 antibodies recognizing either N- or C-terminal epitopes. We found that PU-bound Hsp90 displayed somewhat greater resistance to trypsin than did the GA-bound protein (Figure 1F). To test whether a similar pattern existed *in vivo*, we used drug beads to affinity capture Hsp90 from SkBr3 cell lysate (Figure 1G). PU-bound Hsp90 was markedly more protected at both major protease cleavage sites, consistent with a more compact structure compared to GA-bound Hsp90.

## GA and PU trap distinct Hsp90 conformations but neither drug promotes N domain dimerization

**Figure 2:**
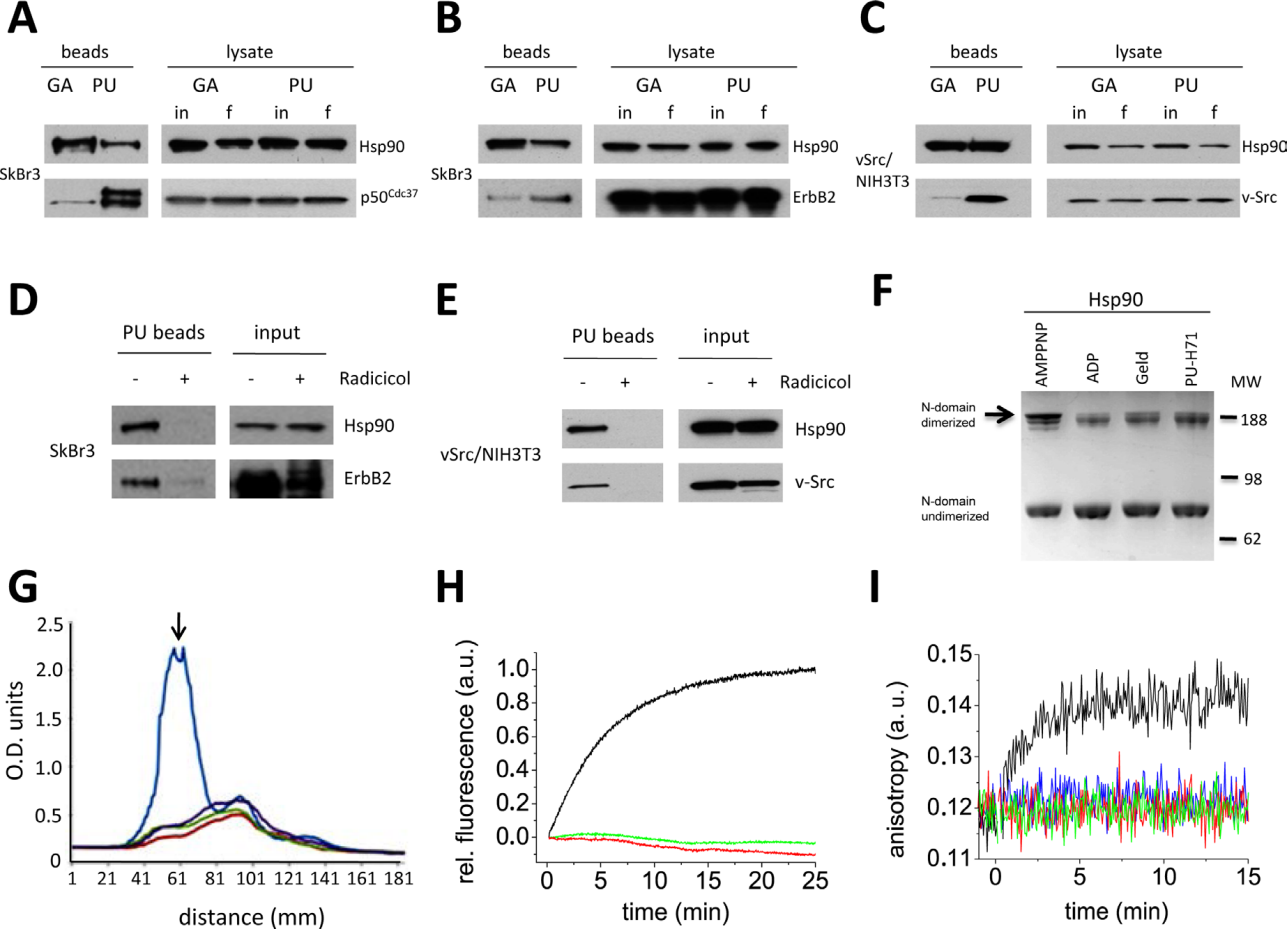
N-terminal Hsp90 inhibitors geldanamycin (GA) and PU-H71 (PU) interact with different sub-populations of N domain undimerized Hsp90 (A-C) Hsp90 was isolated from tumor cell lysates using drug-conjugated agarose beads. Hsp90 levels before (input, in) and after (final, f) chemical precipitation were monitored by Western blot. PU, but not GA, preferentially associates with Hsp90 bound to the co-chaperone p50^Cdc37^ (A), and the oncoprotein kinase clients ErbB2 (B) and v-Src (C). (D and E) Pre-incubation of intact cells with the chemically distinct N-terminal inhibitor radicicol prior to PU-bead precipitation blocked Hsp90 and oncoprotein binding, confirming specificity of the PU-bead pulldowns. (F-I) N domains of GA- and PU-bound Hsp90 are in close proximity but are not dimerized. (F) N domain dimerization of yeast Hsp90 determined by DMS cross-linking in the presence of AMPPNP, ADP, GA (Geld) and PU (PU-H71). (G) Average densitometry scans of (F) were normalized against total intensity (AMPPNP, blue tracing; ADP, red tracing; GA, green tracing; PU, purple tracing). The peak representing the slowest migrating band (identified by the black arrow) indicates the degree of N-terminally dimerized Hsp90 protein. (H) Neither GA nor PU alone induce significant conformational changes that would correspond to N-terminal dimerization as assayed by FRET. Time-dependent FRET induced by addition of either 2 mM ATPγS (black tracing), 50 μM GA (green tracing), or 50 μM PU (red tracing) to 400 nM Hsp90 is shown. (I) Fluorescence anisotropy detection of p23 binding to yHsp90. Fluorescein-labeled p23 was excited at 490 nM and emission was recorded at 520 nM. p23 binding to Hsp90, which requires N domain dimerization, is only detectable after addition of 2 mM AMP-PNP (black tracing), but not after addition of 10 μM GA (green tracing), 10 μM PU (red tracing), or 100 μM PU (blue tracing).

Based on measurements using purified proteins, GA and PU display similar Hsp90 binding efficiencies (GA Kd = 1.35 μM, PU Kd = 0.78 μM). Therefore, the disparate binding profiles of GA and PU in cells, shown in Figure 1, likely are influenced by factors in the cellular environment. Supporting this hypothesis, a recent study found that PU-beads readily affinity purify a population of Hsp90 bound to clients [12], in contrast to GA beads [23]. We sought to verify this discrepancy by screening for the presence of well-documented Hsp90 clients in our pulldown system. Kinases comprise a large part of the Hsp90 clientele [24] and Hsp90-dependent kinases are delivered to the chaperone by p50^Cdc37^, an Hsp90 co-chaperone that maintains Hsp90's ATP lid in an open conformation to facilitate client loading [25-28]. Therefore, we investigated the presence of p50^Cdc37^ in GA- and PU-bound Hsp90 populations. This co-chaperone co-precipitated to a much greater degree with PU-bound than with GA-bound Hsp90 (Figure 2A). Further examination of affinity captured Hsp90 complexes revealed that PU-bound Hsp90 was associated to a much greater degree than was GA-bound Hsp90 with the client kinases ErbB2 (Figure 2B) and v-Src (Figure 2C). Pre-treatment of intact cells with radicicol, a chemically unrelated N-terminal Hsp90 inhibitor that occupies the same nucleotide binding pocket as does PU (and GA), abrogated both ErbB2 and v-Src pulldown by PU-beads (Figure 2D, E), confirming specificity. These data support the likelihood that PU, in contrast to GA, can access and efficiently trap Hsp90 species bound to p50^Cdc37^ and client kinases.

Because PU-bound Hsp90 remains associated with client proteins, we asked whether the drug may allow some degree of N domain dimerization, which is thought to confer more avid client binding to Hsp90 [29-30]. First, in order to visualize N domain dimers of purified Hsp90 we performed cross-linking with dimethyl suberimidate (DMS) in the presence of AMP-PNP (a non-hydrolyzable ATP analog), ADP, GA or PU, and we assessed N domain dimerization by Western blot. Only AMP-PNP promoted significant N domain dimerization (Figure 2F). Averaged densitometric scans of these results are depicted in Figure 2G, where the arrow identifies the degree of N-terminally dimerized Hsp90 protein.

To confirm these results, we performed fluorescence resonance energy transfer (FRET) analysis using purified yeast Hsp90 with FRET probes on the N domain of one protomer and on the M domain of the other protomer. N domain dimerization is a prerequisite for FRET to be generated by the close opposition of these two domains. As in the previous experiment, only the stable ATP analog ATPγS induced a FRET signal (Figure 2H). In contrast, neither GA nor PU was able to do so. Finally, using fluorescence anisotropy generated by fluorescein-labeled p23 binding (which requires N domain dimerization [31]), we found that neither GA nor PU supported p23 binding to Hsp90, again in contrast to AMP-PNP (Figure 2I) and consistent with the impact of these drugs on p23 binding to Hsp90 in cells [23, 32]. This was confirmed by FRET analysis with donor-labeled p23 and acceptor-labeled yeast Hsp90, where only after addition of AMP-PNP was an increase of acceptor fluorescence detectable (data not shown). These data agree with our earlier observation that p50^Cdc37^ was found in PU-bead pulldowns of Hsp90, since ATP lid closure, which is antagonized by this co-chaperone [27], initiates the conformational changes necessary for N domain dimerization and establishment of the p23 binding motif [31, 33-34]. Taken together, our findings definitively show that neither GA nor PU binding to Hsp90 is compatible with N domain dimerization, suggesting that the client-interacting Hsp90 species trapped by PU-beads must be in an ‘open’ (e.g., N domain undimerized) conformation.

**Figure 3:**
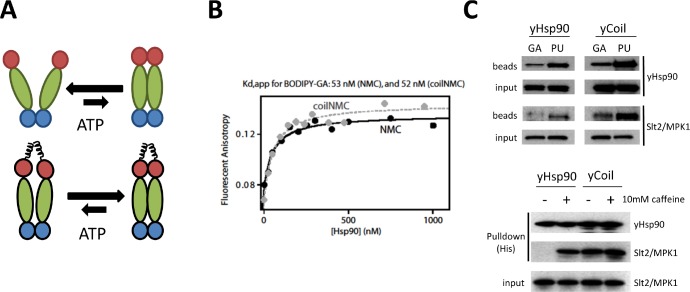
Both GA and PU can access Hsp90 whose N domains are in close proximity but are not dimerized (A) Model of the proposed effect on Hsp90 conformation of enforced N domain proximity achieved by adding coiled-coil domains to the Hsp90 N-terminus. (B) Coiled-coil yeast Hsp90 (coilNMC) has a similar affinity for GA as wild-type Hsp90 (NMC), as determined by BODIPY-GA displacement. (C, Top) Coiled-coil yeast Hsp90 (yCoil) robustly binds both GA and PU, and allows for client capture with GA-beads. (Bottom) Coiled-coil domains strengthen yHsp90 client (Slt2/MPK1) binding.

To explore further the range of open Hsp90 conformations accessible by PU and GA, we assessed drug binding to purified yeast Hsp90 protein engineered with an N-terminal coiled-coil “clamp” to enforce N domain proximity (but not N domain dimerization) and to limit the range of available N domain undimerized conformations ([35], cartoon depicted in Figure 3A). Although our protease sensitivity data suggested a model in which GA preferentially traps a more open, extended form of Hsp90 compared to PU, we found that equilibrium binding of GA to both wild-type (NMC) and coiled-coil (coilNMC) yeast Hsp90 protein was similar, based on fluorescence anisotropy of BODIPY-GA (Figure 3B), suggesting that GA can also access a more compact Hsp90 conformation, as previously proposed [36]. Further, we found that both GA- and PU-beads efficiently isolated coiled-coil Hsp90 from yeast lysates, compared to wild-type Hsp90 (Figure 3C, top panel, ‘yHsp90' blots).

The yeast mitogen-activated protein kinase Slt2/MPK1, which is involved in cell wall integrity, has been found associated with Hsp90 only after activation with 10 mM caffeine, presumably because association of the inactive kinase with Hsp90 is too weak to be detectable with standard pull-down techniques [37]. Pull-down of wild-type Hsp90 from yeast lysate confirmed these results (Figure 3C, bottom panel, left two lanes). In contrast with these data, PU-beads, but not GA-beads, co-precipitated detectable amounts of non-activated Slt2/MPK1 with wild-type Hsp90 from yeast lysate (Figure 3C, top panel, left two lanes, ‘Slt2/MPK1' blots), suggesting that as in mammalian cells PU is able to trap and stabilize a subpopulation of the open Hsp90 conformation displaying stronger affinity for weakly interacting Hsp90 client proteins. Interestingly, coiled-coil Hsp90 also associated efficiently with non-activated Slt2/MPK1 (Figure 3C, bottom panel, right two lanes), and both PU-beads and GA-beads pulled down non-activated Slt2/MPK1 associated with coiled-coil Hsp90 (Figure 3C, top panel, right two lanes). These findings are consistent with a model of Hsp90/client interaction in which the compact, N domain undimerized Hsp90 conformation (stabilized by the presence of an N domain coiled-coil motif but normally comprising a minor percentage of the open wild-type Hsp90 population at steady-state) possesses unexpected avidity for weakly interacting client proteins, and this conformation is preferentially accessible to PU and perhaps to other Hsp90 inhibitors.

## Phosphorylation state of Hsp90 contributes to inhibitor selectivity

**Figure 4:**
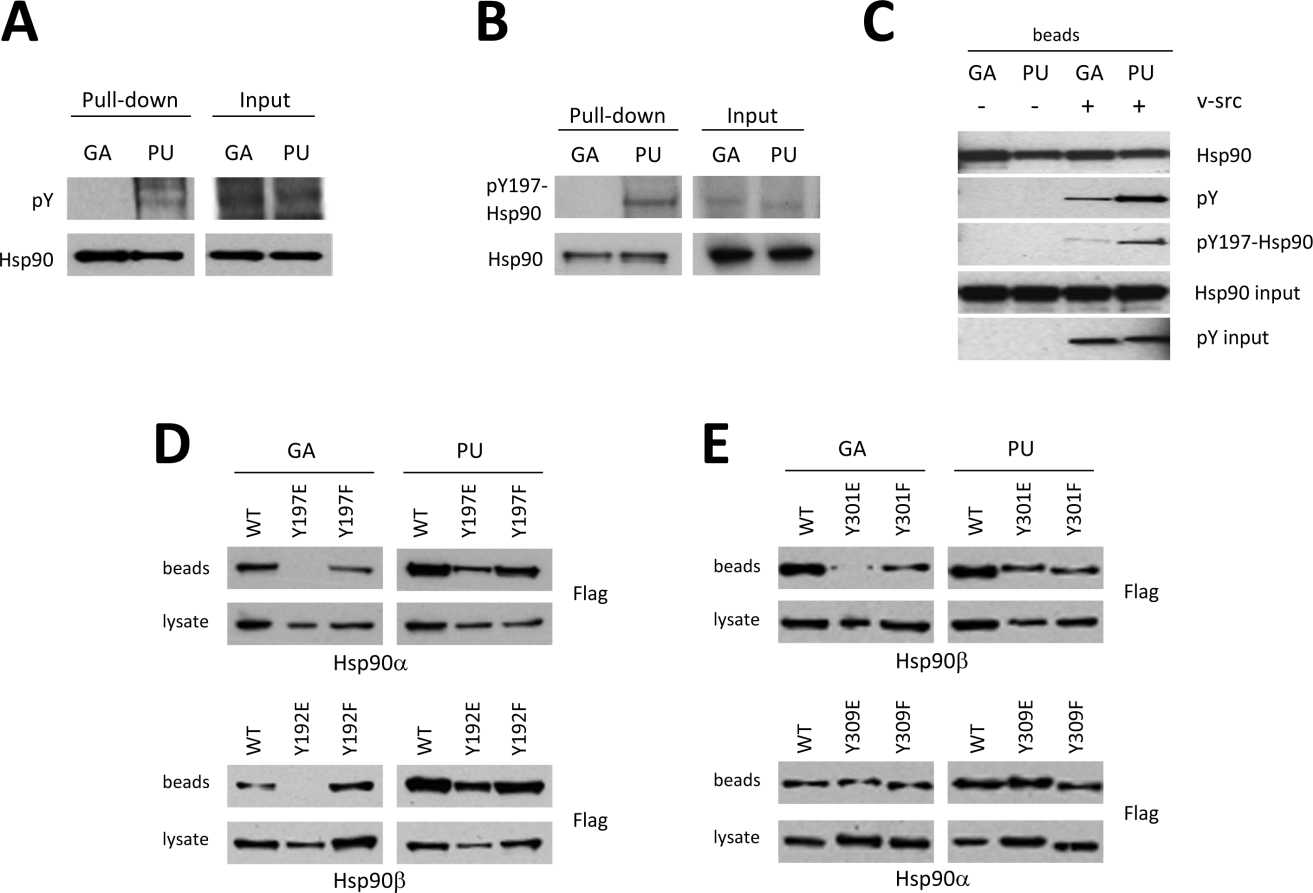
Post-translational modification of Hsp90 governs drug specificity (A) Endogenous Hsp90 from 293T cells was isolated with GA or PU beads and Western blotted with antibody to pan-phospho-tyrosine. (B) Endogenous Hsp90 was subjected to GA and PU beads as in (A) and probed with an antibody specific to phospho-tyrosine 197 (Hsp90α). (C) *In vitro* v-Src-dependent tyrosine phosphorylation of Hsp90 bound to either GA or PU beads. (D, E) Phosphorylation can influence both the binding and isoform specificity of Hsp90 inhibitors. Flag-tagged wild-type and phospho-mimetic mutant Hsp90α and Hsp90β in cell lysates were subjected to GA and PU beads. Phosphorylation of Y197 (Hsp90α) or Y192 (Hsp90β) reduced GA binding without affecting PU binding (D). Phosphorylation of Y301 affected GA binding to Hsp90β only; binding of phospho-Y301-Hsp90β to PU, or phospho-Y309-Hsp90α to either GA or PU was unchanged compared to wild-type (E).

While these data suggest that GA or PU binding preferences depend on the conformational state of Hsp90, the influence of cellular environment on these parameters implies that posttranslational modification of Hsp90 may contribute to inhibitor selectivity [13-20]. Protein phosphorylation/dephosphorylation is one of the most dynamic regulatory processes in cells and growing evidence suggests that Hsp90 phosphorylation contributes to Hsp90 regulation in eukaryotes ([14-17, 20]; also see Phosphosite Plus website). Thus, we asked whether Hsp90 phosphorylation status affected recognition by GA- or PU-beads. First, we observed that, in the presence of the potent phosphotyrosine phosphatase inhibitor bpV(phen), PU-beads captured tyrosine-phosphorylated Hsp90 with greater efficiency than did GA-beads (Figure 4A). Next, using a site-specific phospho-Hsp90 antibody (anti-pY197, numbering for human Hsp90α [20]), we confirmed that Hsp90α phosphorylated endogenously on Y197 bound to PU- but not to GA-beads (Figure 4B). Likewise, when purified Hsp90α was subjected to *in vitro* phosphorylation with purified v-Src protein prior to challenge with GA- or PU-beads, we observed a similar preference of GA-beads for non-phosphorylated Hsp90 (Figure 4C).

Since the phosphorylation status of Y197 affects GA binding more severely than it does PU binding, we queried the ability of phosphomimetic (Y197E) and non-phosphorylatable (Y197F) Hsp90α mutants to bind to GA- and PU-beads. Since this tyrosine residue is conserved in Hsp90β (Y192), we also examined drug-bead binding to the equivalent Hsp90β mutants (Y192E and Y192F). In agreement with our previous data, interaction of either phosphomimetic mutant (Hsp90α-Y197E or Hsp90β-Y192E) with GA-beads was undetectable, while GA-beads bound the non-phosphorylatable mutants (Hsp90α-Y197F and Hsp90β-Y192F) similarly to wild-type Hsp90 (Figure 4D). In contrast, binding of PU-beads to both Hsp90α and Hsp90β was minimally affected by these mutations, consistent with the hypothesis that the phosphorylation status of this tyrosine residue may be an important determinant of GA binding but has minimal impact on PU binding.

Finally, we examined whether the phosphorylation status of a second conserved tyrosine (Y301 in Hsp90β and Y309 in Hsp90α) affected drug binding. Although PU binding was not influenced by mutation of this residue in either Hsp90 isoform, phosphomimetic mutation of this tyrosine in Hsp90β (Y301E) markedly reduced GA binding. Interestingly, we did not observe similar effects for Hsp90α, where neither phosphomimetic nor non-phosphorylatable mutation (Y309E and Y309F, respectively) altered GA binding (Figure 4E). These observations suggest that the phosphorylation status of this residue may provide isoform specificity to GA (but not PU) binding.

Taken together, our data suggest that, although N domain small molecule Hsp90 inhibitors sharing the same binding pocket and possessing comparable affinities for Hsp90 similarly inhibit the chaperone cycle at an early stage, they may preferentially access distinct populations of the open conformation and are not equally affected by Hsp90 posttranslational modification. As proof of principle, we have shown here that PU-H71 accesses a broader range of open Hsp90 conformations than does geldanamycin and it is less affected by Hsp90 phosphorylation, consistent with its broader and more potent cellular activity [12]. Importantly, we have shown also that phosphorylation can provide isoform specificity to Hsp90 inhibitor binding. Although *in vitro* reconstituted systems have provided valuable information concerning Hsp90 structure and interactions with co-chaperones, client proteins and N domain inhibitors, such studies cannot assess the influence of cellular environmental factors on these parameters, including the potentially complex impact of the chaperone's numerous and dynamic posttranslational modifications on drug binding. As our current data clearly demonstrate, the cellular milieu strongly influences these parameters in ways not predicted by *in vitro* observations, thus providing an additional level of complexity to optimal Hsp90 targeting *in vivo*.

## MATERIALS AND METHODS

### Mammalian cell culture and lysis

The HER2+ breast cancer cell line SkBr3 was obtained from the American Type Culture Collection. The embryonic kidney epithelial cell lines HEK293-H and HEK293-T were purchased from Invitrogen. NIH-3T3 fibroblasts expressing v-Src were a kind gift from O. Sartor (Tulane University) [38-39]. Cells were cultured in McCoy's 5A (SkBr3) or DMEM (293-T, 293-H) media supplemented with 10% fetal bovine serum (Life Technologies). All cell lines were propagated at 37°C in an atmosphere containing 5% CO_2_. After washing in PBS, adherent cells were lysed in TNES buffer (50 mM Tris pH7.5, 1% NP40, 2 mM EDTA, 100 mM NaCl) with freshly-added protease and/or phosphatase inhibitor tablets (Roche). Total soluble protein concentration was determined using the BCA protein quantification kit (Thermo Scientific Pierce) following the manufacturer's instructions.

### Transfection

293-H cells were transfected with pcDNA3.1-Flag-tagged Hsp90 constructs (wild-type Hsp90α, wild-type Hsp90β, Hsp90α-Y197E and -Y197F, Hsp90β-Y192E and -Y192F, Hsp90α-Y309E and –Y309F, and Hsp90β-Y301E and –Y301F) using FuGENE6 or XtremeGENE9 transfection reagents (Roche) following the manufacturer's instructions. After incubation for 18 h, cells were lysed as above and assayed for protein expression and drug-conjugated agarose binding.

### Yeast growth and lysis

Yeast strain pp30 (*MAT a*, *trp1-289, leu2-3,112, his3-200, ura3-52, ade2-101, lys2-801, hsc82KANMX4, hsp82KANMX4*) [40] carrying either wild-type Hsp82 (NMC), or Hsp82 with N-terminal coiled-coils (coilNMC) [35] were used in this study. Yeast were grown on YPD (2% (wt/vol) Bacto peptone, 1% yeast extract, 2% glucose, 20 mg/liter adenine). Selective growth was on dropout 2% glucose (DO) medium with appropriate amino acids [14]. The medium pH was adjusted to 6.8 with NaOH before autoclaving. 5-Fluoroorotic Acid (5-FOA) plates were prepared as previously described [41]. Yeast lysis was carried out as previously described [14]. The cell wall integrity MAP kinase pathway was stimulated by 10 mM caffeine.

### Chemical precipitation

GA-affinity beads and PU-affinity beads were synthesized as previously described [39, 42-43]. All bead conjugates were washed in lysis buffer, added to 200 - 400 μg total protein in 400 μl total volume, and rotated at 4°C for 1 h. The volume of bead conjugates used was determined by pilot experiments to insure equal amounts of Hsp90 precipitated from each cell line used, and volumes varied between batches of beads. However, in all experiments, bead volumes were 20 - 40 μl (50% slurry). Following incubation, bead conjugates were washed 3 times in lysis buffer and subjected to Western blot analysis.

### Western blot analysis

Total or chemically-precipitated protein samples were separated by SDS-PAGE, transferred to nitrocellulose membrane, blocked, and probed overnight with primary antibodies against total Hsp90 (Enzo Life Sciences), ErbB2 (Thermo Scientific), v-Src (Santa Cruz Biotechnology), His (Qiagen), Slt2/MPK1 (Santa Cruz Biotechnology), N-terminal Hsp90 (Thermo Scientific Pierce), C-terminal Hsp90 (Enzo Life Sciences), pan-phospho-tyrosine (Millipore), Hsp90 phospho-Y197 (Cell Signaling Technology), or FLAG (Sigma Aldrich). After incubation with appropriate secondary antibodies, detection was performed using the SuperSignal chemiluminescence detection system (Thermo Scientific Pierce) following the manufacturer's instructions. Films were scanned using a Bio-Rad GS-700 imaging densitometer equipped with Quantity One software.

### Trypsin proteolysis

Drug-bound Hsp90 (chemically precipitated recombinant human Hsp90α or Hsp90 chemically precipitated from SkBr3 cell lysate and then washed in 500 mM NaCl to remove adventitiously associated proteins) was digested with increasing concentrations of tosyl phenylalanyl chloromethylketone (TCPK)-treated trypsin (Worthington). Reactions were incubated on ice for 6 min and digested fragments were visualized by immunoblotting with both N- and C-terminal anti-HSP90 antibodies.

### Fluorescent GA binding

BODIPY-GA was synthesized as described [44]. A 100 nM stock was reduced with 15 mM dithiothreitol in assay buffer (20 mM Tris pH 7.5, 5 mM magnesium chloride, 100 mM potassium chloride, 0.01% Nonidet P-40) for 1 hour at 4°C in order to convert GA to the high-affinity hydroquinone form. From this stock, samples with 5 nM BODIPY-GA and purified Hsp90 proteins (NMC or coilNMC) at concentrations ranging from 0-1000 nM were prepared in assay buffer with 15 mM DTT. These samples were incubated at 23°C for 1 hour prior to measurement of fluorescent anisotropy. Anisotropy measurements were made in a 0.3 cm path length cuvette on a PTI QM-4SE spectrofluorometer with excitation set to 488 nm and emission at 510 nm. The apparent Kd was determined by fitting the anisotropy measurements to a simple binding model.

### Fluorescence resonance energy transfer (FRET)

Methods for performing FRET analysis were similar to those described previously [33].

### p23 anisotropy

Fluorescein-labeled p23 was excited at 490 nM and emission was recorded at 520 nM. Measurements were performed in 40 mM Hepes, 150 mM KCl, 5 mM MgCl_2_ pH 7.5 with 50 nM p23-Fluorescein and 500 nM yHsp90 at 30°C. Addition of 2 mM AMP-PNP was used as a positive control to promote N domain dimerization, thereby creating a binding site for p23.

### Hsp90 cross-linking

Hsp90 N domain dimerization assay using the chemical cross-linking agent DMS was performed as described previously [34].

### *In vitro* protein kinase assay

Human Hsp90α was N-terminally tagged with hexahistidine using pRSETA plasmid. 1 mg of bacterially expressed and purified protein was incubated with 100 μl of Ni-NTA agarose (Qiagen). 200 ng of baculovirus expressed and purified v-Src (SignalChem) was used, and kinase reactions were performed as previously described [45].
